# PARP Inhibitors in Advanced Prostate Cancer in Tumors with DNA Damage Signatures

**DOI:** 10.3390/cancers14194751

**Published:** 2022-09-29

**Authors:** Ciara S. McNevin, Karen Cadoo, Anne-Marie Baird, Stephen P. Finn, Ray McDermott

**Affiliations:** 1 Department of Histopathology and Morbid Anatomy, Trinity Translational Medicine Institute, Trinity College Dublin, D08 W9RT Dublin, Ireland; 2 Department of Medical Oncology, St. James’s Hospital, D08 NHY1 Dublin, Ireland; 3 School of Medicine, Trinity Translational Medicine Institute, St. James’s Hospital, D08 W9RT Dublin, Ireland; 4 Department of Medical Oncology, Tallaght University Hospital, D24 NR0A Dublin, Ireland; 5 Department of Medical Oncology, St. Vincent’s University Hospital, D04 YN26 Dublin, Ireland

**Keywords:** metastatic castrate resistant prostate cancer, PARP inhibitors, homologous recombination repair, BRCA

## Abstract

**Simple Summary:**

This review paper seeks to summarize the current literature on the role of PARP Inhibitors in Advanced Prostate Cancer in tumors with defects in genes associated with DNA damage repair. It will give particular attention to the role of PARPi in tumors with non-BRCA DNA damage repair genes. The aim of this review is to summarize the literature on PARPi and their activity treating BRCA and non BRCA tumors with DNA damage signatures.

**Abstract:**

Since 2010, significant progress has been made in the treatment of metastatic castrate resistant prostate cancer (mCRPC). While these advancements have improved survival, mCRPC remains a lethal disease, with a precision medicine framework that is lagging behind compared to other cancers. Poly (ADP-ribose) polymerase (PARP) inhibitor (PARPi) studies in prostate cancer (PCa) have focused primarily on the homologous recombination repair (HRR) genes, specifically BRCA1 and BRCA2. While homologous recombination deficiency (HRD) can be prompted by germline or somatic BRCA1/2 genetic mutations, it can also exist in tumors with intact BRCA1/BRCA2 genes. While the sensitivity of PARPi in tumors with non-BRCA DNA damage signatures is not as well established, it has been suggested that genomic alterations in DNA damage repair (DDR) genes other than BRCA may confer synthetic lethality with PARPI in mCRPC. The aim of this review is to summarize the literature on PARPi and their activity treating BRCA and non BRCA tumors with DNA damage signatures.

## 1. Introduction

While 5-year survival for people with localized PCa in the US, as reported in 2022, remains greater than 99%, this decreases to 31% in the metastatic disease setting [1]. In 2021, PCa remained the second leading cause of cancer related death in men in the US and Europe [2,3] making it a significant public health burden. However, the treatment landscape for mCRPC, has witnessed huge advancements over the past 15 years. These developments have contributed to a reduction in the mortality rate by 52% in the last twenty years from 39.3 per 100,000 cases in 1993 to 18.8 per 100,000 cases in 2017 [4].

Traditionally the use of molecularly targeted therapy in PCa focused on the inhibition of oncogenic drivers (e.g., androgen receptor (AR)) and first-generation AR targeted agents (flutamide, bicutamide and nilutamide) dominated the treatment paradigm since the seminal paper by Huggins et al. over fifty years ago [5,6]. Since then, the approval of additional agents such as next generation AR signaling inhibitors (ARSi) (abiraterone [7], enzalutamide [8], apalutamide [9], darolutamide [10]), chemotherapies (docetaxel [11], cabazitaxel [12]), Radium 223 [13] and Sipuleucel-T [14] have contributed to improved outcomes in advanced PCa. The approval for the first tumor agnostic treatment that for high levels of microsatellite instability (MSI-H) or mismatch repair-deficient (MMRD) solid tumors (pembrolizumab) included PCa [15,16]. This finally pushed mCRPC into the age of precision medicine, albeit later than other cancer subtypes. While the use of Poly(ADP-ribose) polymerase (PARP) inhibitors (PARPi) for patients with DNA damage repair (DDR) mutations is well established for ovarian [17], breast [18] and pancreatic cancer [19], the first PARP inhibitors (PARPi) approved for PCa by the FDA occurred in 2020. This was the result of two trials investigating Olaparib [20] and Rucaparib [21], which demonstrated improved survival outcomes for people with mCRPC with somatic or germline DDR (see Table 1).

While PARPi are most frequently associated with defective BRCA1/2 genes causing HRD, studies have shown that people with other germline and somatic DNA damage repair (DDR) mutations (such as ATM, ATR, CHK1, CHK2, DSS1, RPA1, NBSI, FANCD2, FANCA, CDK12, PALB2, BRIP1, RAD51B, RAD51C, RAD51D and RAD54) may also respond to treatment with PARPi [22,23]. However, the list of defective genes causing HRD is nuanced and far from complete. It has been acknowledged that sequencing for panels of HR related genes may fail to identify a subset of HR deficient cases. Focusing on specific DNA damage signatures or ‘DNA scars’ that result from HR is an encouraging approach to evaluate HRD [24].

Identification and understanding of these genetic nuances could provide much needed granularity on molecular stratification of PCa with the aim of guiding future clinical trial eligibility and treatment selection. This could increase the percentage of people that may benefit from PARPi and open up treatment options to a heavily pretreated cohort. Once established in the metastatic setting for HRR genes, it would be iterative to explore therapeutic selection options in early-stage disease, an evolution that has been demonstrated in the breast and ovarian cancer space [25,26]. Yet, the practical utility of mutations in DDR genes as markers for PARPi sensitivity is not as well described (other than BRCA1/2) and existing data describing the impact of DDR defects is limited and conflicting [27,28,29,30,31,32]. This review aims to provide an update on the activity of PARPi in PCa tumors with DNA damage signatures. Specifically, it will focus on the potential for synthetically lethal interactions between PARPi and non-BRCA DNA damage repair genes in mCRPC.

**Table 1:** Practice Changing Trials and Drug Approvals for mCRPC.

Year	Trial	Drug Class	Study Treatment	Control	(*n*)	Pretreated Chemo (c) ADT (h) (ARSi)	Sequence Approved by FDA	HR for Death (95% CI)	Biomarker
2004	Tax 327 [11]	Chemo	Docetaxel	Mito + P	1006	(h)	1st line	0.76 (0.62–0.94)	N/A
	SWOG 9916 [33]	Chemo	Docetaxel + Estramustine	Mito + P	674	(h)(c)	1st line	0.80 (0.67–0.97)	N/A
2010	TROPIC2 [34]	Chemo	Cabazitaxel	Mito + P	755	(h)(c)	2nd line	0.70 (0.59–0.83)	N/A
	IMPACT [35]	IO	Sipuleucel-T	Placebo	512	(h)(c)	1st line	0.77 (0.61–0.98)	N/A
2011	NCT00321620 [36]	BMA	Denosumab	ZA	1904	(h)	1st line	1.03 (0.91–1.17)	N/A
	COU-AA-301 [7]	Chemo	Abiraterone + P	Placebo + P	1195	(h)	2nd line	0.65 (0.54–0.83)	N/A
2012	AFFIRM [37]	ARSi	Enzalutamide	Placebo	1199	(h)(c)	2nd line	0.63 (0.53–0.75)	N/A
2013	COU-AA-302 [38]	ARSi	Abiraterone + P	Placebo + P	1088	(h)	1st line	0.75 (0.61–0.93)	N/A
	ALSYMPCA [36]	Radio-pharmaceutical	Radium-223 + SOC	SOC	921	(h)	1st line	0.70 (0.58–0.83)	N/A
2014	PREVAIL [8]	ARSi	Enzalutamide	Placebo	1717	(h)	1st line	0.71 (0.60–0.84)	N/A
2017	KEYNOTE 028 [16]	IO	Pembrolizumab	Placebo	23	(h)(c)(ARSi)	3rd line	N/A	MMR/MSI
2019	TITAN [39]	ARSi	Apalutamide	Placebo	1207	(h)(c)	2nd line	0.67 (0.51–0.89)	N/A
	ARAMIS [10]	ARSi	Darolutamide	Placebo	1509	(h)	1st line	0.71 (0.50–0.99)	N/A
2020	PROFOUND [20]	PARPi	Olaparib	Placebo	387	(h)(c)(ARSi)	2nd line	0.55 (0.29–1.06)	HRD *
2021	TRITON2 [40]	PARPi	Rucaparib	Placebo	115	(h)(c)(ARSi)	3rd line	N/A	BRCA1/2 Mutation

Mito: Mitoxantrone; P: Prednisolone; IO: Immunotherapy; BMA: Bone Modifying Agent; ZA: Zoledronic Acid; SOC: Standard of Care; MMR: mismatch repair genes; MSI: microsatellite instability; HRD *: deleterious or suspected deleterious germline or somatic HRR gene-mutated.

## 2. DNA Repair, Cellular Pathways and Synthetic Lethality

### 2.1. DNA Damage Repair Pathways

Cells have evolved a complex signaling network of repair processes known as DDR to rapidly detect and repair cells against constant intrinsic and extrinsic insults. These vital pathways safeguard the repair of DNA to ensure their ability to survive by repairing single strand breaks (SSBs) and double strand breaks (DSBs) [41], and thus maintain genomic integrity. Mechanisms for repairing DSBs include single stranded annealing (SSA), alternative NHEJ, homologous recombination (HR) and non-homologous end joining (NHEJ). Single strand break repair (SSBR), mismatch repair (MMR), nucleotide excision repair (NER) and base excision repair (BER) are mechanisms that ensure SSBs are repaired.

**Figure 1:**
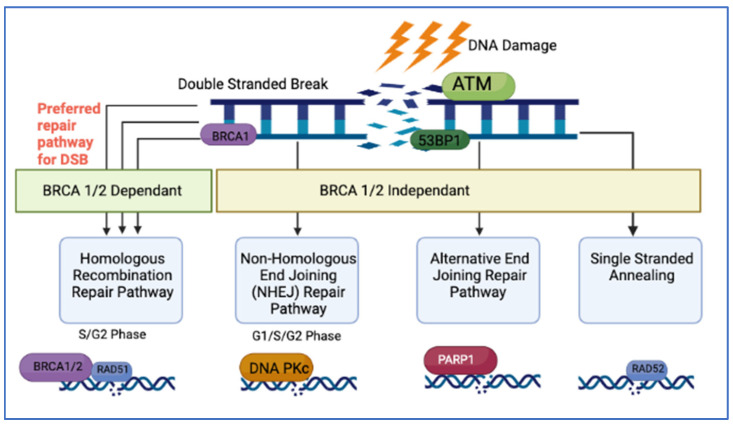
Repair Pathways for DSBs.

HR, considered a BRCA1/2 dependent pathway, is the preferred repair pathway for DSB for BRCA1/2 proficient cells [42,43]. In this pathway, HR exploits the duplicate genetic data located on the homologous sister chromatid. During the S-phase of DNA replication HR uses the sister chromatid as a reference to restore nucleotides of damaged DNA in the instance of replication fork stalling [44,45]. BRCA1 promotes end resection with BRCA2 loading RAD51, which in turn facilitates error free repair by catalyzing the homology search on the sister chromatid [46]. The presence of sister chromatids is limited to S and G2-phase, therefore cells are unable to utilise HR throughout the whole cell cycle. The absence of an complete repair template in G1-phase means cells mainly depend on the BRCA1/2 independent NHEJ for the repair of DSBs [42]. BRCA1/2 deficient cells, which regulate S and G2-phase also get shunted towards BRCA1/2 independent pathways [43]. Pathway selection is regulated by the presence or absence of phosphorylation of 53BP1 by the ATM serine/threonine kinase that initial recognizes DSB. The decision as to whether initiate end-resection (i.e., HR pathway) or block end-resection (i.e., end-protection resulting in NHEJ), is determined by either the presence of 53BP1 or by BRCA1 and CtIP [42]. (Figure 1).

Genomic instability demonstrated in malignant cells drives the accumulation of mutations. This in turn prompts the development of tumor heterogeneity [47]. Mutations in DDR genes can compromise a cells integrity, the significance of which is emphasized by numerous human syndromes (i.e., Lynch syndrome and hereditary nonpolyposis colorectal cancer). DNA repair alterations have been described in approximately 20% of mCRPC (See Table 2 and Table 3 below). The most frequent alterations are in HR genes such as BRCA2, BRCA1 and ATM, and can be present at either the somatic (tumor) or germline (genetic) level [48,49]. It is well established that deleterious germline mutations of BRCA1 or BRCA2 can compromise HR. This has clinical relevance, as mutations in BRCA2 are recognized as a risk factor for men to develop PCa (8.6 fold in men ≤ 65 years). Mutations in BRCA1 also showed an amplified risk for men (3.5 fold) [50,51]. Further, men with a germline BRCA alteration are reported to have poorer clinical outcomes, more commonly having disease which has spread beyond local involvement than men without a BRCA mutation [51,52]. Therefore, that is little doubt that these mutations can translate to pathology in men with PCa. Other genes in the same pathways (such as PALB2, RAD51B, RAD51C, RAD51D, XRCC2, XRCC3, and BARD1) have also been linked to an increased cancer risk, due to the adoption of alternative routes of DSB repair via more error-prone pathways. This results in the buildup of damage and ultimately expediting tumorigenesis [43].

Genomic instability caused by mutations in DDR genes, while a threat to cell integrity that can promote carcinogenesis, can also be exploited by targeted therapies that use these fragilities in DDR processes to promote cancer cell apoptosis [53]. Treatments that overwhelm this sophisticated DDR machinery in cancer cells, can provide a valuable therapeutic opportunity. Chemotherapy and radiotherapy are two traditional exploiters of this concept, often inflicting DNA damage to halt cancer cell proliferation or trigger cancer cell apoptosis [53]. This ‘double hit’ phenomenon whereby a cell is more susceptible to an insult (such as PARPi) when it already has a genomic defect (such as a BRCA1/BRCA2 mutation) is known as ‘synthetic lethality’ [54].

### 2.2. Synthetic Lethality and PARP

**Figure 2:**
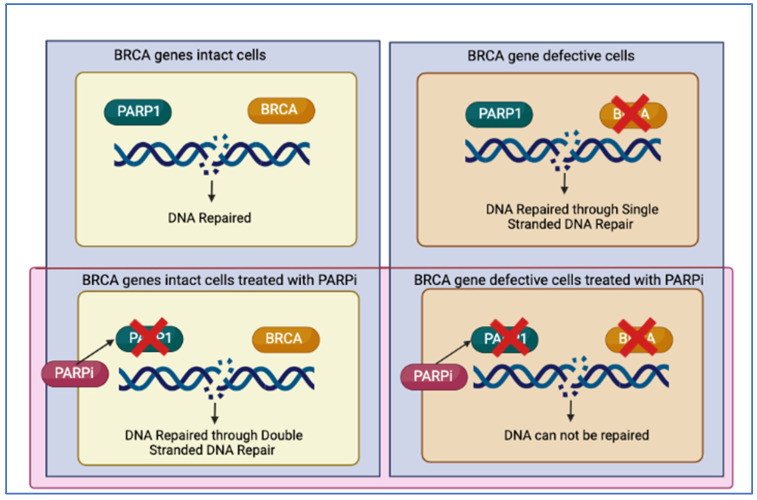
Synthetic lethality as the mechanism behind PARPi treated BRCA deficient cells leading to irreparable DNA damage.

PARP proteins are a group of 17 ADP-ribosyltransferases enzymes that use nicotinamide adenine dinucleotide (NAD+) to create polymers of ADP ribose units (PAR) on specific proteins through covalent attachments in a reaction called polyADP-ribosylation (PARylation). PARP1, the founding member of the PARP family, is the most abundant and ubiquitously expressed PARP enzyme. While PARP1 is the best characterized [55], PARP2 and PARP3 also play a role in this process [56]. DNA damage is swiftly identified through the conserved N-terminal DNA-damage detecting and binding domain of PARP. PARP1 is recruited to these SSBs and DSBs in genomic DNA, which starts its catalytic activity by 500-fold [57]. PARP1 then cleaves nicotinamide adenine dinucleotide (NAD+) and moves the resulting ADP-ribose onto itself or other target proteins. This process is a post-translational modification, which auto-activates PARP and other DNA-repair enzymes in response to DNA damage [58]. This is an essential activity to recruit PAR-binding factors to the damaged site involved in DNA repair [53]. Cancer cells harboring a defective BRCA1/2 gene, are unable to use the HRR pathway to repair DNA damage, and therefore require PARP proteins to aid single stranded DNA repair. If PARP proteins are inhibited by a therapeutic (i.e., A PARPi), the DNA cannot be repaired, and cell death occurs [59] (Figure 2). When two insults simultaneously cause cell death as descrbied above, while one change alone is nonlethal, this is the concept of synthetic lethality [43]. A traditional illustration of this is demonstrated by a study in which BRCA2 mutations were reported to be more likely to respond to carboplatin-based chemotherapy compared to PCa in which BRCA2 was intact in cases of CRPC [60]. In other words, treatment with platinum-based chemotherapy causing genomic strand breaks in tumor cells with impaired HR may be translated in a synthetic lethality [51].

PARPi are an established synthetic lethal partner of defective BRCA genes [43] and they have been shown in clinical trials to have greater efficacy in individuals with a genetic mutation than those without genetic defects, (described in greater detail in Section 4). However, PARP proteins intersect with many different cellular stress and genome integrity pathways [61] and therefore have multiple opportunities to harness activity within the repair pathways. Identification of HRD tumors with functioning BRCA1/BRCA2 genes, is clinically relevant as translational and clinical studies indicate that these tumors may benefit from HRD associated therapy, i.e., PARPi/Platinum [20,60]. Yet, primary resistance to these therapies has been observed a subset of in PCa with alterations in HRR. Conversely, there are cases without documented alterations in HRR genes who show a extended response [62]. This has been adopted and clinically demonstrated in high grade serous ovarian cancer (HGSOCa) where the PRIMA Trial (NCT02655016) demonstrated a Progression Free Survival (PFS) benefit with Niraparib for women with platinum-responsive advanced ovarian cancer in first-line monotherapy maintenance treatment regardless of biomarker status [63]. This led to the first PARPi to be approved by the FDA for people without a BRCA mutations in April 2020 [64].

### 2.3. The Concept of BRCAness and Mutational Signatures

HRD with impaired fork repair machinery and the resulting susceptibilityto DNA damaging agents are known as ‘BRCAness’. This is a phenotype that replicates the impact of a BRCA1/BRCA2 defect on HRR [65]. Cells identified as displaying this characteristic rely on more error-prone repair pathways [42]. HR ensures the error-free repair of DSB. When this process is malfunctioning specific DNA scars accumulate in the genome. These are the result of error-prone repair of DSBs that occur due to replication stress. There are identifiable HRD-induced DNA abnormalities or scars varying from single nucleotide variation (SNV) to larger scale genomic rearrangements [24]. The definition of BRCAness has been extended to include replication fork protection (RFP) and regulatory mechanisms that cause synthetic lethality with PARPi [42,65]. The rising accessibility of next-generation sequencing (NGS) data from cancer cells has facilitated the identification of distinct mutational signatures associated with BRCAness [42].

The first description of mutational signatures was reported when technological advances in the 1960′s led to the description of how UV damage principally results in thymine–thymine or cytosine–cytosine or cytosine-thymine variations, favorably appearing at pyrimidine dimers (i.e., C > T or CC > TT DNA mutations at dipyrimidine sites) [66]. DNA sequencing advances allowed for the analysis of distribution of somatic mutations across the cancer genome. These revealed distinctive arrangements or patterns known as mutational signatures [67]. The vast majority of somatic mutations in a cancer genome are not thought to be pathological and are known as passenger mutations. Only a small percentage of the recognized variants are associated with cancer development [42,68].

Alexandrov et al. (2013) through algorithmic analysis of millions of signatures across thousands of cancers extracted 21 distinct validated mutational signatures with probable associations (i.e., Age, UV light, smoking, etc.) [68]. Across distinct cancer subtypes, the Catalogue Of Somatic Mutations In Cancer (COSMIC) Signature 3 (cSig3) was closely associated with BRCA1/2 mutations. Further, most samples habouring a BRCA1/2 mutations displayed a significnat contribution from CSig3. Intriguingly, several cases with a notable contribution from CSig3 did not habour a BRCA1/2 alteration. This suggests that other mechanisms or abnormalities of other genes may also create it. Of note, CSig3 was not identified in PCa in Alexandrov’s analysis [42]. Signature 6 (cSig6) was characterized by a pattern of indels, often termed ‘microsatellite instability’, which is a distinguishing characteristic of cancers with defective DNA mismatch repair. This description was reinforced by a strong association with Signature 6 and the inactivation of DNA mismatch repair (MMR) genes in colorectal cancer. An underlying mutational process was not identified for many mutational signatures. It has been hypothesized that these may be due to currently uncharacterized defects in DNA maintenance.

Sztupinszki et al. (2020) building on Alexandrov’span-cancer study, investigated mutational signatures associated with HRD in PCa that are not associated with germline or somatic BRCA1 or BRCA2 mutations [24]. A total of 311 samples from 240 cases, the majority of which were localized PCa cases (n = 215), underwent WGS. Their study demonstrated aside from deletions in BRCA1 or BRCA2, elimination of any of a number of other key HR genes (RAD51C, XRCC2, XRCC3, PALB2, RAD54) also induces the same mutational signatures typically associated with HRD. They demonstrated that in 5–10% of localized PCa cases, WGS data displayed HRD associated mutational signatures even without loss of function mutations in BRCA1/2 or other canonical homologous recombination genes. This proved that HRD-patterns were demonstrated in men with PCa who did not harbor germline or somatic mutations in BRCA1/2 or other known HR-related genes [24]. This has significant translational implications as the contribution of cSig3 to SNVs may be reasonably good at predicting platinum and PARPi sensitivity [69]. De Sarkor et al. (2021) investigated mutational signatures in PCa using WES from 418 cases examining the pattern of genomic aberrations and mutation signatures in non BRCA HRD genes, the results of which are discussed further in Section 4.2 [62].

## 3. Genomic Heterogeneity in PCa

While therapeutic advancements over the past 15 years have been welcomed, an appreciation of the heterogeneity of the genomic landscape of PCa has been slow to be recognized and people with PCa have historically been treated as a genetically homogenous group. PCa is highly heritable with 57% of the inter-individual variation in risk attributed to genetic factors [70], with family history recognized as a primary risk factor [71]. Yet, despite years of linkage studies, identifying the genes associated with PCa predisposition has been a challenge [72]. Initially, genomic profiling of PCa was drawn from material from unselected prostatectomies and genetic defects were considered to be rare [73]. Germline mutations in BRCA2 were underappreciated as a risk factor driving hereditary PCa with only 1–3% of unselected localized diagnoses harboring BRCA2 germline mutations [74]. Prospective genomic classification of fresh biopsy sections in those with mCRPC has historically been incomplete due to challenges in attaining satisfactory tumor material, particularly from bone biopsies [75,76]. However, genomic heterogeneity in PCa and clinical relevant genomic drivers have now been further described [77] thanks to the formative work by Pritchard [49], Robinson [78], Quigley [79], Abida [48] and Dall ‘Era [80] as summarized in Table 4. Additionally, Castro [81] and Mateo [82] linked genomics with clinical outcomes in two further informative studies.

Robinson et al. (2015) described clinical sequencing of somatic aberrations in 150 men with metastatic mCRPC, evaluating a group of 38 selected genes [78]. In 19.3% of cases mutations in BRCA2, BRCA1 and ATM genes were observed. A total of 63% of men were identified as having a mutation in AR, not an unexpected finding in metastatic hormone resistant disease. In non-AR pathways, targetable alterations were also identified PI3K pathway (49%), DNA repair pathway (19%), RAF kinases (3%), CDK inhibitors (7%) and the WNT pathway (5%). Adding to these somatic mutations, pathogenic germline mutations were seen in 8% of men with mCRPC. Overall just under 90% of individuals in the study held a clinically targetable defect in either somatic or germline (Table 4). Pritchard (2016) collated the data from seven separate case series of men with advanced PCa between the UK and the US looking at 20 germline genetic mutations [49]. Unlike Robinson et al. who focused on somatic mutations, Pritchard reported incidence rates of germline genetic mutations in men with metastatic PCa as follows; BRCA2 (5.35%); CHEK2 (1.87%), ATM (1.59%) and BRCA1 (0.87%). Collectively Pritchard describes DDR germline defects representing 25% of these alterations, with BRCA2 being by far the most prominent (44%), followed by ATM (13%), CHEK2 (12%) and BRCA1 (7%).

Quigley (2018) obtained fresh-frozen core biopsies of metastases from 100 mCRPC participants and performed whole-genome and transcriptome sequencing to provide detailed analysis of structural variations that disturb primary regulators in PCa [79]. Pathogenic activating AR mutations or amplifications of AR were observed in 85% of mCRPC samples. Somatic mutation frequencies in BRCA2, ATM, and BRCA1 were congruous with earlier reports in mCRPC (Table 2). Inactivating germline alterations in DNA repair genes (BRCA2 and ATM) were reported in 4% of samples.

Abida (2019) performed an analysis of genomic and transcriptomic profiles through whole-exome sequencing on 444 biopsies from 429 people with mCRPC, including a description of clinical outcomes [48]. More than 20% of participants harbored mutations in DNA repair, PI3K, cell-cycle or epigenetic pathway genes. Single-nucleotide variants (SNVs) in the frequently effected genes were thought to be pathologic in most cases with a high fraction of oncogenic mutations in AR, TP53, PIK3CA, BRCA2, PTEN, APC, and CDK12. Genomic alterations in BRCA2, BRCA1 and ATM were reported at 11%, 7% and 1.9%, respectively.

Castro et al. (2019) screened men at diagnosis of mCRPC for germline DDR mutations in 107 genes [81]. The main aim was to evaluate the influence of ATM/BRCA1/BRCA2/ PALB2 germline mutations on cause-specific survival (CSS) in men with mCRPC. One of the secondary aims stated was to evaluate men habouring germline DDR defective genes and their response to treatments. Median CSS was 10 months shorter in ATM/BRCA1/BRCA2/PALB2 carriers than in noncarriers yet the difference was not significant (23.3 vs. 33.2 months; *p* = 0.264; Hazard Ratio (HR), 1.32; 95% Confidence Interval (CI), 0.81 to 2.17). While the the study did not meet its primary end point, it did reveal that CSS was halved in men with an identified BRCA2 gene defect compared with noncarriers, a finding that reached statistical significance (median, 17.4 vs. 33.2 months; *p* = 0.027; HR, 2.10; 95% CI, 1.07 to 4.10). CSS was also significantly different when BRCA2 carriers were compared with other non-BRCA2 gDDR carriers (median, 33.8 months; *p* = 0.048). Multivariable assessments recognized BRCA2 as an self standing prognostic factor for CSS in mCRPC (HR, 2.11; 95% CI, 1.06–4.18) [81].

Mateo et al. (2020) evaluated 470 treatment-naive PCa biopsies and for 61 cases of whom went on to develop mCRPC, whole-genome sequencing (n = 52) was performed. TP53 (27%) and PTEN (12%) and DDR gene defects (BRCA2 7%; CDK12 5%; ATM 4%) were commonly detected [82]. RB1 loss in the primary tumor had a worse prognosis as previously described. This study suggests that lethal PCa is enriched for DNA repair defects from diagnosis, before developing castration resistance, which has implications for treatment and testing sequencing.

Dall et al. (2020) assessed 24 germline and somatic DNA repair genes in 944 men with both early and advanced PCa [80]. A total of 152 participants of the 944 men (16%) harbored a genetic alteration (either germline or somatic) in at least one DNA repair genes. The most frequently mutated genes were BRCA2 (11.4%) and ATM (5.8%), followed by MSH6 (2.5%) and MSH2 (2.1%). Pathogenic BRCA1 variants were identified at a frequency of 1%. The majority of BRCA 1 and BRCA2 genes were truncating mutations (80% and 70%, respectively), and all were monoallelic with allele frequencies of 0.4 to 0.8, pointing towards a germline mutation.

The prevalence of somatic and germline aberrations has been summarized in Table 3 and Table 4, respectively. Of interest, these studies display variability among results. For example, 85% of mCRPC samples carried either pathogenic activating AR mutations, amplifications of AR, or putative AR enhancer region amplifications in Quigley et al. analysis, which is higher than the 63% of cases identified as carrying these AR alterations in Robinson et al. Further, inactivating germline alterations were present in the DNA repair genes (BRCA2 and ATM) in 4% of samples in Quigley et al., a slightly lower frequency than the approximately 10% frequency observed in Pritchard study. This is partly due to a variance in germline versus somatic mutations being tested for and method of interrogation of samples. For example, Robinson et al. adopted whole-exome sequencing versus the Quigley et al. study who adopted a whole-genome and transcriptome sequencing approach. It is acknowledged that the exome represents less than 2% of the genome. Further data regarding prevalence of genetic mutations can be garnered from relevant clinical trials, which screened for germline and somatic mutations (Table 5).

## 4. Clinical Impact of DNA Damage Signatures in mCRPC

### 4.1. DNA Repair Pathways and Clinical Trials with PARPi

TOPARP-A (NCT01682772) was the formative phase II clinical trial investigating treatment of mCRPC with Olaparib with prospectively identified genetic biomarkers [22]. Overall, participants with mutations in DNA repair genes had a better response rate to Olaparib, with 14 of 16 participants (88%) deemed ‘biomarker positive’ reported as demonstrating a response. Conversely only 6% of the ‘biomarker-negative’ participants were reported as having a response. TOPARP-B (NCT01682772) a randomized phase II trial for men with PCa that had progressed to mCRPC, came after by TOPARP-A [87]. TOPARB B reported a response to Olaparib in 47% of participants with DNA damage response gene aberrations. The TOPARP trials were privotal in demonstrating efficacy of Olaparib when used to treat men with mCRPC possessing certain DDR genetic aberrations. It further suggested that PARP inhibitions men benefit men with PCa that do not habour a BRCA mutation.

PROfound was a phase III randomized control trial that compared Olaparib with an ARSi, either abiraterone or enzalutamide, in two cohorts of men with mCRPC. Cohort A (n = 245) was made up of men with BRCA1/BRCA2 or ATM gene alterations while Cohort B (n = 142) contained men with 12 other pre-determined DNA Damage repair genes alterations [20]. The PFS was notably longer in the treatment group compared to the control group (5.8 m vs. 3.5 m; HR, 0.49; 95% CI, 0.38 to 0.63; *p* < 0.001). This survival advantage was more marked in Cohort A.(7.4 m vs. 3.6 m); HR for progression or death, 0.34; 95% (CI), 0.25 to 0.47; *p* < 0.001). Based on these results the FDA approved Olaparib as a treatment for men with mCRPC with germline or somatic deleterious or suspected deleterious HRR, who have progressed following prior treatment with Enzalutamide or Abiraterone.

TRITON2 examined the efficacy Rucaparib, another PARPi, for men with mCRPC. They examined men with deleterious BRCA mutation (germline and/or somatic) who had previously received an ARSi and a taxane-based chemotherapy [21]. The population included 115 participants who had a deleterious germline (n = 44) or somatic (n = 71) BRCA1 (n = 13) or BRCA2 (n = 102) alteration. The confirmed overall response rate (ORR) was 43.5% (95% CI, 31.0% to 56.7%; 27 of 62) and was 50.8% (95% CI, 38.1% to 63.4%; 33 of 65) per independent radiology review and per investigator assessment was 50.8% (95% CI, 38.1% to 63.4%; 33 of 65), respectively. The results of this study have led to its recent approval by the FDA.

A secondary analysis of TRITON2 was published by Abida et al. in 2021. This study evaluated 78 participants focusing on non-BRCA DDR genes. Namely this included ATM (n = 49), CDK12 (n = 15), CHEK2 (n = 12), and other DDR genes (n = 14) [84]. Among those assessed, radiographic and PSA responses were observed in a limited number with an alteration in ATM (2/19 (10.5%) and 2/49 (4.1%), respectively), CDK12 (0/10 (0%) and 1/15 (6.7%), respectively), or CHEK2 (1/9 (11.1%) and 2/12 (16.7%), respectively). This contained no radiographic or PSA responses in 11 men with confirmed biallelic ATM loss or 11 men with ATM germline mutations. Rucaparib efficacy was further reported in cases of men with mutations in the DDR genes namely PALB2, FANCA, BRIP1, and RAD51B.

TALAPRO-1 is a phase II trial investigating men with progressive mCRPC with known DDR likely to sensitize to PARPi (ATM, ATR, BRCA1/2, CHEK2, FANCA, MLH1, MRE11A, NBN, PALB2, RAD51C) to receive the PARPi, Talazoparib. Primary endpoint was defined as objective response rate (ORR) [85]. After a median follow-up of 16.4 months, the ORR was 29·8% (31 of 104 patients; 95% CI 21.2–39.6).

GALAHAD is a phase II trial assessing PARPi Niraparib in men with progressive mCRPC and DNA-repair gene defects (DRD) as defined as having biallelic alterations in BRCA1/2 (BRCA), ATM, FANCA, PALB2, CHEK2, BRIP1, or HDAC2 [86]. The authors used composite response rate (CRR), defined as ORR, conversion of circulating tumor cells to <5/7.5 mL blood, or ≥50% decline in PSA. As of 23 May 2019, 165 participants were enrolled, 81 of whom had biallelic DRD (46 BRCA genes and 35 non-BRCA genes). In BRCA genes, ORR was 41% and CRR was 63%. The median duration of objective response was 5.5 months (range: 3.5–9.2). 7/12 BRCA responses were ongoing. Median radiographic progression-free survival (rPFS) and OS in BRCA were 8.2 and 12.6 months, respectively. Objective response was noted in 2/22 pts in non-BRCA cochort, (both had FANCA) and CRR was 17%.

### 4.2. Specific Non-BRCA Biomarkers in DNA Repair Pathways and Mutational Signatures

Ovarian and breast cancer studies suggest that single genomic parameters lack sensitivity and specificity to identify HRD and accurately reflect treatment outcomes in all cases [88]. In the PCa space, while the PROfound study indicated that subgroups selected for treatment based on genetic stratification, showed greater clinical benefit to treatment compared to a genetically distinguishable subgroup, identifying certain HRD associated gene mutation is an imperfect predictor of clinical response to treatment [20,21]. This suggests more work is needed to understand the role individual mutations. The TRITON2 study and TALAPRO-1 results are providing much needed granularity of specific genes response to treatment (Table 6 and Table 7). That said, non-BRCA individual genetic mutations are imperfect as sole predictors of ‘BRCAness’ and subsequent selectors of candidates for HRD directed therapies. Advances in cancer genomes should enable the adoption of mutational signatures over individual genomic mutations. Below we outline the relationship between non BRCA individual genetic mutations in PCa and their association with cSig3.

#### 4.2.1. ATM Gene

ATM somatic mutations are present in approximately 5.8 to 7.3% of mCRPC (Table 2) and approximately 1.6–1.9% of men with mCRPC have a germline ATM mutation (Table 3). Moreover, the FDA have approved Olaparib for individuals harboring a deleterious or suspected deleterious germline or somatic mutation in ATM. Yet, those with ATM have demonstrated inconsistent responses to PARPi in clinical trials in PCa. In the first PARPi clinical trials, TOPARP-A, PARPi appeared to be efficacious in participants with an ATM with 4/5 participants with deleterious ATM mutations having response to Olaparib [22]. In TOPARP-B the response was less impressive with those harboring an ATM mutation having a composite OR of 36.8% (7/19). Yet, in the PROfound trial, men with ATM mutations were grouped with participants with BRCA1/2 mutations, and this group showed superior response compared to Cohort B (described above). However, when interrogated men with ATM alterations did not do any better than those in the control group. The Triton study which included 49 cases with an identified ATM mutation, reported a radiographic response and PSA response post treatment with Rucaparib of just 10.5% and 4.1%, respectively [84]. The TALAPRO [85] study reported of 18 cases with ATM mutations with a reported ORR of 11.8% (2/17). Abida et al. described mutations in ATM, and predicted them to be oncogenic in nearly 60% of cases, with the rest being missense mutations of unknown significance [48]. In the De Sarkar et al., which evaluated the relationship between ATM and cSig3, only one of 15 tumors with ATM-BAL was CSig3 positive (7%) and 3 (20%) showed positivity when classified using the authors integrated assessment of HRRd (iHRD). Carreira (2021) further interrogated the samples in the TOPARP-B trial, assessing whole-genome sequencing, immunohistochemistry (IHC) and immunofluorescence (IF) assays. Of the 21 men who had an ATM mutation identified 16 were somatic mutations and 5 were germline mutations. ATM loss of expression by IHC was associated with longer rPFS (median 5.8 months vs. 3.7 months) and OS (median 17.4 months vs. 10.3 months). This suggests the loss of ATM protein identied by IHC is associated with a better outcome [89]. While people with mCRPC harboring a ATM mutations have not consistently responded to PARPi in clinical trials in PCa, cell line models suggest that treating ATM altered tumors with both a PARP inhibitor and an ATR inhibitor may be more efficacious as compared with PARP inhibition alone [90].

#### 4.2.2. PALB2

Somatic mutations and germline mutations in PALB2, a binding partner and nuclear localizer of BRCA2, in mCRPC were found in approximately 6% and 0.4%, respectively (Table 2 and Table 3). In the TOPARP-B trial, those with PALB2 mutations achieved composite OR in four of seven cases, and four of six achieved PSA responses, indicating potential benefit from PARP inhibition. One case, habouring biallelic PALB2 aberrations, reported a a durable response that lasted for 39 weeks. In TRITON2, two of two participants with PALB2 alterations experienced PSA responses. One of these cases achieved a partial radiographic response, while the second had a 47% reduction in tumor volume [35]. In the PROfound study, Olaparib did appear to provide some benefit in men with PALB2 mutations. Studies in breast cancer have shown that small samples (n = 3) with germline nonsense/frameshift variants in PALB2 have exhibited elevated Signature 3 activity [91]. Similarly, in PCa, small samples of PALB2 mutation or copy loss (n = 2) were associated with CSig3 positivity [62]. While PALB2 is a potential effective biomarker of response to HRD directed therapy, clinical qualification of low-prevalence biomarkers is challenging. Given the small numbers, the contribution of PALB2 mutations must be considered with caution as other modes of BRCA1/BRCA2 inactivation or other genes related to the HR pathway could underlie the observations in these trials [92].

#### 4.2.3. CHEK2

The was a low prevalence of CHEK2 mutations in trials in men with mCRPC, notably TOPARP (n = 2), PROfound (n = 17), and TRITON2 (n = 2) trials were low. In TOPARP (n = 2), one with a CHEK2 mutation demonstrated a response to Olaparib, while one was a non responder. TOPARP-B, reported a CHEK2 alteration participant achieving a PSA decrease of 50%. Preliminary reports from the TRITON2 data report one participant achieving a radiological response and a PSA reduction, with another also achieving a PSA reduction. In the Profound trial no meaningful response data can be derived as CHEK2 mutations were spread between Cohort A (n = 5) and Cohort B (n = 12) and often occurred in conjunction with another known pathogenic driver mutation (i.e., BRCA2). Similar to the above, without higher participant numbers, it will be difficult to draw conclusions. More studies in those with CHEK2 mutated tumors are warranted. In one breast cancer study, where 4 germline pathogenic variants in CHEK2 were identified, CHEK2 was not associated with a high level of cSig3 [90]. In PCa specifically, tumors with biallelic CHEK2 loss (n = 6) were uniformly CSig3 negative [62].

#### 4.2.4. FANCA

FANCA and its sister FANC proteins activate the BRCA repair pathway and play a significant role in DDR. It is hypothesized therefore people with mutations in the FANC family could benefit from PARPi therapies. In the preliminary TRITON2 data, one participant with a monoallelic truncating mutation (of 4 participants with FANCA alterations) had complete radiographic and PSA responses. While in the TOPARP-B, one participant with a nonsense FANCA mutation achieved a PSA response. Breast cancer studies did not show a linkage between FANCA-FANCN and cSig3 positivity [91], and studies in PCa did not derive any meaningful conclusions, as frequencies of cSig3 positivity were significantly greater compared to the cohort of reference tumors [62].

#### 4.2.5. RAD51

There are several RAD51-related genes, including RAD51B, RAD51C, RAD51D, DMC1, XRCC2 and XRCC3, which work together with BRCA2 to maintain replication fork stability and independently to promote fork reversal in the process of repairing DSBs in HRR (Figure 1). Somatic and mutations in RAD51 were found in approximately <1% and 0.4%, respectively as outlined in Table 2 and Table 3 above. Participation numbers of those with RAD51 mutations in completed PARPi clinical trials in PCa has been low. From preliminary results from the TRITON 2 study included 3 participants with a mutation from the RAD51 family (1 each had a RAD51, RAD51B, or RAD54L alteration) [84]. The participant with a RAD51B alteration had a partial radiographic and a PSA response, both ongoing. From breast cancer studies it is suggested that inactivation of RAD51C can lead to HRD, as cases with inactivation of RAD51C exhibited high levels of Signature 3, however the numbers were small (n = 2). De Sarkar et al. reported that there was one case of biallelic RAD51C loss associated with CSig3 positivity [62].

#### 4.2.6. CDK12

Inactivation of CDK12 delineates a distinct immunogenic genomic pattern in PCa which does not exhibit DNA mutational signatures linked to HRD [93]. From De Sarkar et al., 21 tumors were identified as having biallelic CDK12 inactivation. After excluding 2 tumors due additional mutations, 1 of 19 CDK12 tumors considered were CSig3 positivity. Yet, there is some evidence that CDK12 loss exhibits synthetic lethality with PARPi [94], though early results of PARPi in CDK12-mutant mPC have identified few responses [20,84].

## 5. Conclusions

Traditionally oncological diagnostic and treatment decisions were predominantly based on tumor morphology, clinical symptoms and the cancer site of origin. However, the advances in cancer genomes, specifically the impact of germline and somatic mutations of BRCA1/2 and the effacy of PARPi in PCa have shifted the treatment paradigm. That acknowledged, prospective genomic characterization of PCa has been met with challenges. The low frequency of actionable genomic alterations in primary PCa has perhaps delayed the appreciation of pathological genetic drivers in PCa, and limited inclusion in clinical trials. Further, prospective genomic characterization of fresh biopsy samples from those living with mCRPC has been restricted due to challenges in obtaining adequate tumor tissue, particularly from bone biopsies [76]. The potential for PARPi treatments in men with mCRPC with germline and somatic mutations of BRCA1 and BRCA2 are well recognized, further studies are required to fully appreciate genomic results in PCa in non BRCA HRD tumors. Even in cases where genomic material is available, the low prevalence of some of these mutations means that further studies are required to derive their clinical significance. Thus, the landscape of genomic alterations in mCRPC disease remains incompletely characterized. In the pursuit of precision medicine approaches, and the low prevalence of these mutations means that further data are required to confirm these findings. Further efforts in understanding sequencing results and utilizing WGS to identify mutational signatures, will advance understanding of cancer etiology with potential implications for prevention and treatment.

## Figures and Tables

**Table 2:** Summary of reported somatic genetic aberrations in mCRPC.

Pathway	Gene	Robinson (n = 150) [15]	Quigley (n = 100) [22]	Abida (n = 444) [23]
AR	AR	62.70%	69.31%	
	AR Enhancer		80.20%	
	ASXL2		6.93%	
	FOXA1	12%	18.81%	
	NCOR1	6.70%	1.98%	
	NCOR2	5.30%	0.99%	
Cell Cycle	CCND1	4.70%	7.92%	
	CDKN1B	4.00%		
	CDKN2A	2.70%	3.96%	
	RB1	9.30%	1.98%	
	TP53	53.30%	56.44%	
Chromatin Modifier	CHD1	8.00%	8.91%	
	KDM6A	3.30%	2.97%	
	KMT2C	12.70%	7.92%	
	KMT2D	2.70%	1.98%	
DNA Repair Pathway	ATM	7.30%	5.94%	5.80%
	ATR			<2%
	BRCA1	0.70%	0.99%	1%
	BRCA2	13.30%	9.90%	11.40%
	BRIP1	4.70%		
	CDK12		2.97%	
	CHEK2			<1%
	ERCC2		2.97%	
	MLH1	0.70%	0.99%	<2%
	MSH2		1.98%	2.10%
	MSH6	2.00%	0.99%	2.50%
	PALB2			<1%
	PRKDC		7.92%	
	RAD51			<1%
ETS	ETS fusions	56.70%		
	ETV1		9.90%	
	ETV4		4.95%	
	ETV5		1.98%	
	ERG		42.57%	
PI3K Pathway	AKT1	1.30%	1.98%	
	PIK3CA	5.30%	0.99%	
	PIK3CB	6.00%		
	PIK3R1	5.30%		
	PTEN	40.70%	44.55%	
WNT Pathway	APC	8.70%	8.91%	
	CTNNB1	4%	5.94%	
	RNF43	2.70%		
	RSPO2	1.30%		
	ZNRF3	2%	3.96%	
RAS/RAS Fusions	RAF1	2.00%		
	BRAF	2.70%	3.96%	
	HRAS		1.98%	

**Table 3:** Summary of reported germline DNA Repair Pathway genetic aberrations in mCRPC.

Pathway	Gene	Pritchard (n = 82) [16]	Castro (n = 68) [24]
DNA Repair Pathway	ATM	1.6%	1.91%
	ATR	0.29%	
	BRCA1	0.9%	0.95%
	BRCA2	5.3%	3.34%
	BRIP1	0.18%	
	CDK12		
	CHEK2	1.9%	3.34%
	ERCC2		0.24%
	MLH1		
	MSH2	0.14%	
	MSH6	0.14%	
	PALB2	0.4%	0.00%
	PRKDC		
	RAD51	0.4%	

**Table 4:** Studies assessing the genomic landscape of PCa.

Author (Year)	Year	(n)	Disease Subtype	Somatic(s) V Germline(g)	Testing Adopted	% Clinically Actionable Aberration
Robinson [78]	2015	150	mCRPC	(s)	Panel of 38 genetic mutations	89%
Pritchard [49]	2016	82/692	mPCa	(g)	Panel of 20 germline genetic mutations	25%
Quigley [79]	2018	100	mCRPC	(g)(s)	WGS	n/a
Abida [48]	2019	444	mCRPC	(s)	WES	>20%
Castro [81]	2019	68/419	mCRPC	(g)	Germline DDR mutations in 107 gene	16.2%
Mateo [82]	2020	470 ^1^/61 ^2^	PCa/mCRPC	(g)(s)	NGS ^1^/WGS ^2^	^1^ 23%
Dall ‘Era [80]	2020	154	PCa/mCRPC	(g)(s)	Panel of 24 genetic mutations (NGS)	16%

^1^ PCa: Prostate Cancer. ^2^ mCRPC: metastatic castrate resistant prostate cancer. mPCa: metastatic PCa. NGS: Next Generation Sequencing. WES: Whole Exome Sequencing. WGS: Whole Genome Sequencing.

**Table 5:** PARPi Trials for Treatment for mCRPC.

Year	Trial	Phase	PARPi	Primary End Point	Genes Included	Testing Method	DDR Gene Aberration Detected/Screened (%)	Key Finding
2014	TOPARP-A [83] NCT01682772	II	Olaparib	Response Rate according to RECIST, PSA or CTC	BRCA2, ATM, BRCA1, FANCA, CHEK2, PALB2, HDAC2, RAD51, MLH3, ERCC3, MRE11, NBN	WES from tumor-biopsy samples; germline WES from saliva samples.	16/50 (32%)	Overall RR: 33% (16/49) RR in HRR positive subgroup: 88% (14/16) PFS: HRR + ve: 9.8 vs. HRR − ve: 2.7 months; *p* < 0.001 OS: HRR + ve: 13.8 vs. HRR − ve: 7.5 months; *p* = 0.05
2019	TOPARP-B [84] NCT01682772	II	Olaparib	Response Rate according to RECIST, PSA or CTC	BRCA1/2, ATM, CDK12,PALB2, CHEK1,CHEK2, ARID1A, ATRX, FANCA, FANCF, FANCG, FANCI, FANCM, MSH2, NBN, RAD50, WRN	NGS of biopsies	161/711 (27%)	RR: Olaparib 400 mg group: 54.3% vs. Olaparib 300 mg group: 39.1% PFS: Olaparib 400 mg 5.5 months vs. Olaparib 300 mg 5.4 months OS: Olaparib 400 mg 14.3 vs. Olaparib 300 mg 10.1 months
2020	PROFOUND [20] NCT02987543	III	Olaparib	Imaging-based PFS	Cohort A: BRCA1, BRCA2, and ATM Cohort B: BRIP1, BARD1, CDK12, CHEK1, CHEK2, FANCL, PALB2, PPP2R2A, RAD51B, RAD51C, RAD51D, and RAD54L	FoundationOne CDx NGS of archival or recent biopsy tissue	778/2792 (28%)	Cohort A + B RR: Olaparib 22.0% vs. ADT 4.0% PFS: Olaparib 5.8 vs. ADT 3.5 months OS: Olaparib 17.5 vs. ADT 14.3 months Cohort A RR: Olaparib 33.0% vs. ADT 2.0% PFS: Olaparib 7.4 vs. ADT 3.6 months OS: Olaparib 18.5 vs. ADT 15.1 months
2020	TRITON2 [21,22] NCT02952534	II	Rucaparib	Response Rate according to RECIST, PCWG3 criteria	First Analysis: BRCA1/BRCA2 Secondary Analysis: ATM, CHEK2, FANCA, PALB2, FANCA, BRIP1, and RAD51B	Foundation Medicine. Germline testing by Color Genomics.	115/78	RR: 44% in participants with BRCA1/2 mutations Confirmed PSA response in 51.1% es in BRCA1/2 group, 1 ntsent with a CDK12 alteration, 1 participant with a BRIP1 alteration, and 1 participant with a FANCA alteration
2021	TALAPRO-1 [85] NCT03148795	II	Talazoparib	ORR	ATM, ATR, BRCA1, BRCA2, CHEK2, FANCA, MLH1, MRE11A, NBN, PALB2, RAD51C	Foundation One CDx™ NGS gene panel test. Saliva sample collection for a germline comparator.	127	Objective RR 29·8% (31 of 104 participants)
2021	GALAHAD [86] NCT02854436 (preliminary results)	II	Niraparib	ORR	Total: 81; (BRCA1/2: 46; non-BRCA: 35) BRCA1/2 (BRCA), ATM, FANCA, PALB2, CHEK2, BRIP1, or HDAC2.	Plasma or tissue-based test	127	RR: BRCA ½ 41% vs. Non-BRCA 9% PFS: BRCA ½ 8.2 vs. Non-BRCA 5.3 months OS: BRCA ½ 12.6 vs. Non-BRCA 14

PCWG3 criteria: Prostate Cancer Clinical Trials Working Group 3 (PCWG3) criteria.

**Table 6:** TRITON2 Study.

Gene	(n)	Radiographic Responses (%)	PSA Responses (%)
BRCA2 + BRCA1	115 (102/13)	43.5%	54.8%
ATM	49	10.5%	4.1%
CDK12	15	0	6.7%
CHEK2	12	11.1%	16.7%
*FANCA*, *NBN*, *BRIP1*, *PALB2*, *RAD51*, *RAD51B*, *RAD54L* ^^	14	28.6%	35.7%

^^ Includes participants with an alteration in *FANCA* (n = 4), *NBN* (n = 4), *BRIP1* (n = 2), *PALB2* (n = 2), *RAD51* (n = 1), *RAD51B* (n = 1), and/or *RAD54L* (n = 1).

**Table 7:** TALAPRO-1 Study: Interim Analysis.

Gene	(n)	^a,b^ ORR, % (Response/n)	^b^ rPFS, Months (95% CI)	^b,c^ Composite Response, % (Response/n)
BRCA2 + BRCA1	46	43.9 (18/41)	9.3 (8.1–13.7)	76.1 (35/46)
PALB2	4	33.3 (1/3)	7.4 (2–7.4)	50.0 (2/4)
ATM	18	11.8 (2/17)	5.5 (1.7–8.2)	27.8 (5/18)
OTHER DDR Genes	18	0	3.7 (1.7–3.9)	11.1 (2/18)

^a^ Measurable soft tissue disease per investigator assessment at screening; ^b^ DDR-deficient population; ^c^ Objective response and/or PSA response ≥ 50% and/or CTC conversion (from CTC ≥ 5 to <5). OTHER DDR Genes: ATR, CHEK2, FANCA, MLH1, MRE11A, NBN, RAD51C.
